# Physical exercise and mitochondrial function: New therapeutic interventions for psychiatric and neurodegenerative disorders

**DOI:** 10.3389/fneur.2022.929781

**Published:** 2022-09-07

**Authors:** Lina Sun, Tianbiao Liu, Jingqi Liu, Chong Gao, Xiaohui Zhang

**Affiliations:** ^1^State Key Laboratory of Cognitive Neuroscience and Learning, Beijing Normal University, Beijing, China; ^2^College of P.E and Sport, Beijing Normal University, Beijing, China; ^3^Department of Clinical Medicine, Key Laboratory of Novel Targets and Drug Study for Neural Repair of Zhejiang Province, Institute of Brain and Cognitive Science, Zhejiang University City College, Hangzhou, China

**Keywords:** mitochondria, exercise, psychiatric diseases, neurodegenerative diseases, adult neurogenesis

## Abstract

Psychiatric and neurodegenerative diseases, including major depression disorder (MDD), bipolar disorder, and Alzheimer's disease, are a burden to society. Deficits of adult hippocampal neurogenesis (AHN) have been widely considered the main hallmark of psychiatric diseases as well as neurodegeneration. Herein, exploring applicable targets for improving hippocampal neural plasticity could provide a breakthrough for the development of new treatments. Emerging evidence indicates the broad functions of mitochondria in regulating cellular behaviors of neural stem cells, neural progenitors, and mature neurons in adulthood could offer multiple neural plasticities for behavioral modulation. Normalizing mitochondrial functions could be a new direction for neural plasticity enhancement. Exercise, a highly encouraged integrative method for preventing disease, has been indicated to be an effective pathway to improving both mitochondrial functions and AHN. Herein, the relative mechanisms of mitochondria in regulating neurogenesis and its effects in linking the effects of exercise to neurological diseases requires a systematic summary. In this review, we have assessed the relationship between mitochondrial functions and AHN to see whether mitochondria can be potential targets for treating neurological diseases. Moreover, as for one of well-established alternative therapeutic approaches, we summarized the evidence to show the underlying mechanisms of exercise to improve mitochondrial functions and AHN.

## Introduction

In recent decades, psychiatric or neurodegenerative diseases have attracted increased attention due to the growing number of patients. Psychiatric diseases such as depression disorder (MDD) and bipolar disorder usually see patients suffering from anxiety or depressive moods and changes in physical and emotional reactions that would be exacerbated by even minor environmental changes (1, 2). As for neurodegenerative diseases such as Alzheimer's disease (AD) and Parkinson's disease (PD), the progressive death of neurons commonly induces irreversible neuronal cognitive deficits, motor disability, and complex behavioral dysregulation (3). The unidentified etiology of those diseases strongly limited the development of drugs to prevent the progress of behavioral abnormality. From the angle of symptomatic treatment, it is urgent and necessary to explore supplementary or alternative medicine for improving brain functions.

Neural plasticity provides the ability for the central nervous system (CNS) to adapt to environmental challenges under physiological and pathological conditions (4). In the adult hippocampus, neural plasticity refers to neurogenesis and synaptic plasticity, both of which perform critical roles in regulating emotional and cognitive behaviors. Adult neurogenesis was widely considered as a structural plasticity through its regulation of the neuronal population in certain brain regions. As for the target of alternative or integrative medicine, improving hippocampal neurogenesis could serve as a key therapeutic paradigm against neurological disorders without a clear pathological mechanism (5–7). For this reason, evaluating the mechanism of adult neurogenesis could help explore an applicable pathway to treat neurological diseases.

Emerging evidence indicates mitochondria have a key function in regulating the activity and fate commitment of stem cells (8). In addition, mitochondria have been recognized as key mediators in response to development of neurological disease (9). Physical exercise is an effective way to prevent chronic diseases, including diabetes, neurodegeneration, and psychiatric disorders (10, 11). Through metabolic regulating, exercise is beneficial to mitochondrial functions. It is noteworthy that mitochondria could be the linker between exercise and neurogenesis. Given this, it is necessary to summarize the effects of mitochondria in neural functions and its roles in disease development. In this review, we summarized the functions of mitochondria to regulate adult hippocampal neurogenesis and its potential regulators. Furthermore, we discussed the linkage role of mitochondria to bridge physical exercise and brain functional improvement.

## Hippocampal plasticity and neurogenesis in neurological disorders

Adult neurogenesis is a temporal-spatial progress composed by the self-renewal fate commitment of neural stem cells (NSCs) as well as the maturation of neural progenitor cells (NPCs). In the hippocampus, neurogenesis provides the regenerative resources to clear panic memory (12, 13). The enhancement of hippocampal neurogenesis was shown to promote pattern separation behavior, which enables animals to discriminate between environmental cues related to stress experience (14). While declined hippocampal neurogenesis commonly results in an elevated fear response, which subsequently manifests as inappropriate, uncontrollable expression of fear in neutral and safe environments (15). These documents highlight the critical role of AHN in regulating antidepressant behaviors. The critical role of hippocampal neurogenesis in depressive moods could also be seen in an animal model of seizures, which was demonstrated to be triggered by antidepressants (16). The seizures animal model showed abnormal increase of adult neurogenesis with upregulated immature neuronal numbers in the hippocampal DG region (17). Another type of neural plasticity besides neurogenesis is synaptic plasticity, which includes synaptogenesis as well as synaptic functions like long-term potentiation (LTP) and pre-synaptic plasticity. Dysregulation of synaptic plasticity was also shown to be related to the development of neurological disorders. Immobilization-stressed mice presented intensified fear memory and enhanced long-term potentiation (LTP) (18). In terms of synaptic plasticity, adult neurogenesis can provide a regenerative resource to prevent the neurodegenerative progress and simultaneously enhance the ability in emotional regulation (19). Promoting the AHN was documented as an effective approach against psychiatric disorders, particularly depression. *In vivo* calcium imaging to record neuronal activity in the vDG (ventral dentate gyrus) demonstrated increased neurogenesis correlated to decreased activity of stress-responsive cells, which are active during attacks or while mice explore anxiogenic environments (20). Through conditional knockout of the Bcl-gene in NSCs, Sahay et al. established that there is enhanced AHN in mice and found that improving AHN was sufficient to prevent behavioral dysfunctions in a depression model (21). Additionally, blocking AHN with temozolomide (TMZ) could also result in the comprised therapeutic effects of antidepressants such as SSRIs (selective serotonin reuptake inhibitors) and ketamine (22, 23). Thus, exploring factors in regulating ANH would offer the new drug targets for treating neurological diseases.

## Mitochondrial function and hippocampal plasticity and neurogenesis

Biological regulation of mitochondria involves multiple aspects, including their metabolism, biogenesis, fission, and fusion dynamics and degradation *via* autophagy. Accumulating evidence has been reported to show that all these biological events participate in the regulation of AHN at different levels or conditions (Table 1). Cell metabolism plays a fundamental role in multiple biological events, including energy supply, cell growth, differentiation, and death. During the self-renewal and differentiation process, stem cells undergo a dramatic metabolic reprogram. At an adult hippocampus, the metabolism pattern of NSCs undergoes the switch from glycolysis to oxidative phosphorylation (OXPHOS) following the process of neuronal differentiation (34). In mature neurons, mitochondrial OXPHOS provides high amounts of energy to meet the requirement of neuronal electrophysiological activities (35, 36). Numerous mitochondrial mediators could be applied as therapeutic targets not only for metabolic regulating but also to improve AHN.

**Table 1:** Mitochondrial biology in regulating AHNs in different aspects.

**Research model**	**Mitochondrial biology**	**AHNs events**	**References**
Normal adult mice	Mitochondrial mass and dynamics	Enhanced neuron maturation	(24)
Linage tracing mice model	Mitochondrial dynamics	Daughter cells directs between self-renew or differentiation	(25)
Drosophila multipotent hematopoietic progenitors (like human mammalian myeloid progenitors)	ROS scavenge	Prevented the differentiation	(26)
Human embryonic stem cells	SIRT1 downregulation	Neuroretinal morphogenesis	(27)
Optic atrophy	Perturbation of inner mitochondrial membrane	Atrophy of retinal RGCs	(28)
Amyotrophic lateral sclerosis	Mitochondrial fragmentation, disruption of ETC, reduced ATP production and oxidative stress	Increase in proliferation in the SVZ but decrease in proliferation in the SGZ	(29, 30)
Stroke model	ETC disruption and impaired ATP production	Increased proliferation and death of neuroblasts	(31)
Alzheimer's disease model		Increased NSCs and immature neurons in hippocampus	(32, 33)

### Mitochondrial metabolism in regulating neurogenesis

Mitochondria have been primarily identified as cellular organelles that provide energy. In neurons, mitochondrial dysfunction is reported to be involved in multiple neurodegenerative or psychiatric diseases (37, 38). Dysregulated AHN induced by abnormal mitochondrial function is one of the main reasons to these diseases. According to the environmental changes, quiescent NSCs in the hippocampus are undergoing extensive changes along with proliferative activity, cellular growth, and synaptic growth. Adult NSCs display astroglia features, including 100% GFAP expression, as well as glycolytic cellular metabolisms pattern (39). Following neurogenesis, mature neurons require high amounts of ATP for their biological functions, such as presynaptic vesicle recycling. Mature neurons integrated in neural circuits are highly dependent on the mitochondrial electron transport chain (ETC) and OXPHOS (40–42). Single cell transcriptomics shows the dramatic upregulating profile of OXPHOS-related genes during the neural lineage commitment of hippocampal NSCs. Moreover, specific ablation of mitochondrial transcription factor A (Tfam) in adult NSCs reproduces multiple hallmarks of aging in the hippocampus, including declined neurogenesis. Such alteration could be reversed by pharmacological enhancement of mitochondrial function (34). The evidence suggests mitochondrial metabolism has a critical role in regulating hippocampal neurogenesis and relative physiological process. Suppressing mitochondrial OXPHOS could also affect the other types of adult stem cells. In hematopoietic stem cell (HSCs), deleting PTEN-like mitochondrial phosphatase Ptpmt1 could lead to defective hematopoiesis with impaired differentiation of HSCs (43). Additionally, the metabolic pattern of cells could shift from mitochondrial OXPHOS to glycolysis during the reprogramming process of the inducible pluripotent stem cells (iPSCs), indicating that mitochondria also act critically in embryonic stem cells (44). Generally, switching of mitochondrial function is commonly associated with the energetic demands of stem cells to meet the requirement of their self-renewal or differentiation. Most neurological drugs that are widely used in clinic reportedly have an effect on metabolic regulation. Indeed, mitochondria are widely reported as the target for improving brain functions. The brain functional recovery drug piracetam was documented to prevent declined neurogenesis *via* promoting mitochondrial metabolism in an aging model (34, 45). Antioxidants could also reserve the functions of mitochondria (46). However, it is noteworthy that exercise may elevate the level of radial oxidative species (ROS), which has been recently declared as a mechanism in antidiabetic effects (47). Since antioxidant and exercise can provide effects similar to those of mitochondria, a certain level of ROS might serve as the “second messenger” to promote the fate commitment of the NSCs at the physiological level (48). Hence, exercise could be an effective way to control the level of ROS into physiological reasonable by promoting mitochondrial OXPHOS.

### Mitochondria dynamics and NSCs behaviors

Mitochondria are constantly varying between being fragmented or filamentous networks to adapt to the requirements of cellular functions. According to different energetic demands of the stem cells stage between self-renewal and differentiation, dynamics alterations of mitochondrial morphology are critical in regulating stemness (49). Mitochondrial elongation commonly occurs in aging skeletal muscle cells with increased mitochondrial fusion protein MFN1/2 and the accumulated mutation of mitochondrial DNA (50). Following the neural commitment of NSCs, mitochondria shift from the elongated morphology to fragmentation. On the cell metabolic level, increasing mitochondrial fragments are associated with enhanced OXPHOS and production of ROS, previously mentioned as the second messenger to stimulate downstream signaling like NRF2 and downregulate Notch1 for lineage commitment determination (48). Sirtuins were also considered as regulators to link mitochondrial dynamics with adult neurogenesis (25). Physical exercise, the well-known upregulator of SIRT3 and lipid metabolism, could enhance adult neurogenesis in an unpredictable chronic stress depression model (51). Hypoxia inducible factor (HIF) signaling also provides the link between oxygen levels and mitochondrial dynamics (52, 53). The activation of the HIF complex under hypoxia ensures that energy demands meet pathological conditions by increasing levels of glycolytic enzymes and inhibiting oxygen consumption (54). Such mechanisms also mediate self-derived neural repair under stress. In the hypoxia condition, activation of HIF induces NSCs proliferation and switched their migration in subventricular zone, which promotes regenerative progress in infarction region (55). HIF deletion, however, can impair the AHN and induce learning and memory deficit (56). Therefore, mitochondrial dynamics-mediated redox/oxidative status plays a key role in regulating AHN.

### Mitophagy in regulating neurogenesis

In starvation conditions, autophagy could be rapidly activated to provides a cell with nutrients to survive (57). The selective autophagy of mitochondria, also known as mitophagy, can be processed such that damaged or unwanted mitochondria require degradation (58). As differentiation of stem cells involves extensive cellular remodeling, autophagy ensures the elimination of unnecessary cellular components to maintain an optimal cellular status. It was demonstrated that pretreatment of antioxidant N-acetylcysteine (NAC) attenuated oxidative stress-induced NSCs' self-renewal disruption by suppressing autophagy signaling mTOR and decreased LC3B-II protein expression (59). In contrast, enhancing autophagy in aged satellite cells prevented the senescence and restored regenerative properties (60). Herein, it could hypothesize that mitochondrial morphology is another effect pathway to regulate mitochondrial dynamics in NSCs. At a physiological level, certain levels of mitophagy might be necessary for controlling the differentiation of adult NSCs. However, there is no systematic evidence that indicates the exact mechanisms of mitophagy to regulate the differentiation and self-renewal of adult NSCs.

## Targeting mitochondria in neurological disease treatment

At the cell level, mitochondrial alterations could be regarded as a hallmark for stem cell differentiation. Consistently, impairment of AHN is a well-established biological hallmark of psychiatric diseases and neurodegeneration at the tissue level. Such a relationship indicates that mitochondria could perform be a therapeutic target for neurological diseases. An increasing number of clinical reports have demonstrated substantial mitochondrial damage could contribute to the development of depression and cognitive impairments. Deletion of mtDNA in a child was associated with mitochondrial disease symptoms and mild–moderate unipolar depression (61). Blood sample measurement of mtDNA in bipolar disorder (BD) and MDD patients also showed a lower mtDNA copy number than in controls (62). Another report demonstrated a significant reduction of mtDNA copy numbers in combat PTSD (63), indicating mtDNA or the mitochondrial mass abnormality could be the general phenomena correlated with psychiatric diseases. On the other hand, mitochondria perform as the therapeutic target to psychiatric diseases. SSRIs (selective serotonin reuptake inhibitors) like the antidepressant fluoxetine could promote mitophagy by increasing colocalization of autophagosomes and mitochondria, which thereby eliminates damaged mitochondria in corticosterone-treated astrocytes (64). McCoy et al. compared high novelty responder rats (HRs), which show highly exploratory behavior in a novel environment as well as remarkable resilience to chronic mild stress, and low novelty responder rats (LRs), which are susceptible to chronic stress. They observed that LRs displayed higher cytochrome c oxidase (COX) activity in the dentate gyrus, prefrontal cortex, and dorsal raphe compared to HRs (65). Apart from selected brain regions, a declining skeletal muscle mitochondrial function in aging adults was also shown to be associated with clinically significant depressive symptoms (66). These lines of evidence support the critical regulatory roles of mitochondria in antidepressant functions.

Environmental factors could also induce psychiatric diseases *via* affecting mitochondrial functions. Glombik et al. reported that maternal stress leads to depression-like behaviors in the offspring of rats; they displayed brain mitochondrial abnormalities, including significant downregulation of Ndufv2 (complex I) (67). Animal studies have suggested that mitochondrial abnormalities were augmented by stress, indicating mitochondria are stress-response modulators and contribute to the stress-induced pathophysiology of psychiatric diseases (68). A possible mechanism might be an enhanced requirement of the neural activity during learning or memory coding, which could induce increased mitochondrial respiration and thereby produce more metabolic products to influence the signaling pathways downstream, e.g., ROS and RNS.

Accumulating evidence suggests that improving mitochondrial functions could help the treatment of neurodegeneration (69, 70). The complex I inhibitor rotenone could be utilized as a PD model for drug development (71). The impairment of complex I was associated with reduced ATP levels, oxidative stress, and calcium-mediated damage in such a pathological model (72). In post-mortem tissue of sporadic AD, scientists found mitochondrial dysfunction is correlated with decreased levels of ATP (73). Growing evidence indicated the medications targeting on mitochondria exert the therapeutic effects to neurodegenerative. Metformin, a type-2 diabetes drug approved by the FDA, was shown to enhance adult neurogenesis and showed promising effects on an animal model of AD and PD (74, 75). Another example is the glycogen-like peptide- (GLP-1) analog. It has been reported that the GLP-1 analog could promote adult neurogenesis and attenuate the behavioral dysfunctions in neurodegenerative disorders including PD and AD (76, 77). Herein, improving mitochondrial functions could also result in protective effects against neurodegeneration.

## Physical exercise and mitochondrial function

Alternative and integrative medicine are increasingly proposed as effective strategies to treat psychiatric and neurodegenerative disorders. Due to safety concerns regarding the tolerability and risk of medications (78), an effective alternative therapy is highly requested to attenuate the behavioral disorders. As for neurodegeneration, early prevention of the diseases is currently the most effective strategy due to the limited effects of drugs to halt or prevent the progress of the neuron death. Physical exercise is widely recognized as being part of a healthy lifestyle partly due to its promotion of the maintenance of lifelong mitochondrial quality control (79). Exercise has been increasingly reported for its improvement of adult neurogenesis in both physiological and pathological conditions (80–82). Exercise improves mitochondrial functions *via* its multiple biological effects. It was demonstrated that exercise promoted the production of brain-derived neurotrophic factor (BDNF) levels and could alter mitochondrial function, neuroplasticity, and the rate of apoptosis in the hippocampus and thereby prevented the occurrence of PTSD (83). In a maternal separation depression model, exercise could alter mitochondrial function, serotonin levels, and the rate of apoptosis (84). Herein, mitochondrial functions perform as the linkage between exercise and its neuroprotective effects.

### Exercise-mediated mitochondrial functions in neurogenic effects

In aged mice, physical exercise significantly increased DRP1 protein levels and elevated the rates of respiration and ROS production in mitochondria, which is suggestive of its potential in improving brain functions *via* its regulating mitochondrial electron transport chain function and dynamics (85). In an animal model of Alzheimer's disease, 1 h of swimming exercise for 6 days/week consolidated the intact of mitochondrial cristae and edges, raised the brain ATP production as well as the number of synapses by regulating the expression of GLUT1 and GLUT3 expression levels (86). Antidepressant action of running was highly correlated with an increase of hippocampal neurogenesis and plasticity (81). Compared with its promotion of NSCs' proliferation, the accelerating effects of exercise have a longer latency period (about 2 weeks) on the maturation of new neurons (87). Moreover, structural magnetic resonance imaging suggested hippocampus and brain cortex growth in schizophrenia patients and healthy controls after the endurance aerobic physical training. This evidence indicates exercise can also serve as a promising candidate for pathophysiology-based add-on interventions for schizophrenia (88). A recent study indicates that free wheel running could promote the activation of the quiescent NSCs in the hippocampus by regulating cellular ROS level (89). Therefore, exercise could engage broad effects of neural functions *via* multiple molecular mechanisms.

### Multiple effects of exercise in brain tissue

Exercise could exert multiple biological effects in additional to its roles in mitochondrial functions. Brain inflammation is another key target of exercise for neural tissue. A recent study showed the systematic regulatory mechanism of exercise influenced adult neurogenesis. Injecting plasma derived from voluntary running mice resulted in elevated density of hippocampal DCX^+^ neurons correlating with improved working memory, which were shown to rely on the inflammatory regulation *via* clusterin (90). This report further suggested the effects of exercise mediated AHN may depend on its effects on the peripheral circulation system. It was indicated that LPS could reduce the number of new neurons in aged but not adult mice, while such dysfunctions could be prevented by free wheel running (91). Exercise could also attenuate the inflammatory response in subjects with depression. A study on 61 university students assigned to 6 weeks of different models of exercise including high-intensity interval training (HIT), moderate continuous training (MCT), or no exercise (CON) suggested that MCT exercise could have a positive effect on the promotion of mental health by decreasing TNF-α level (92). Neuroinflammation has been suggested to negatively affect adult neurogenesis, and physical exercise could promote AHN by buffering the inflammation response in neural tissue (93). The activation of microglia mediated the proinflammatory factors, including interleukin-6, TNF-α, ROS, and nitric oxide, which all have anti-neurogenic properties (94). Table 2 summarizes the recent evidence in support of the effects of exercise, showing different patterns of mitochondrial biology as well as neuronal functions (Table 2). However, limited evidence has shown the possible role of mitochondria during exercise and their ability to mediate the functions of neural tissue, particularly adult NSCs.

**Table 2:** Functional impacts of exercise on neuronal mitochondrial fitness/health.

**Exercise model**	**Impacts to mitochondria**	**Impacts to neural tissue**	**References**
Wheel running	Promoted autophagy/lysosome system	No direct evidence	(95)
High-intensity exercise	Activated partial mitochondrial biogenesis	Promoted AHN, attenuated the inflammation	(96)
Regular running exercise	Activated POMC neuronal mitohormesis	Induced the hypothalamic mediated thermogenesis	(97)
Treadmill exercise	Increase mitochondrial biogenesis and OXPHOS level	Possible protective effects to PD animal model	(98)
Treadmill exercise	Prevented mitochondria-mediated caspase-dependent apoptotic pathways	Suppressed neural apoptosis in aging model	(99)
Voluntary exercise	Increased oxygen consumption and ATP production *via* oxidative phosphorylation	Improved dopaminergic functions in PD model	(100)
Low-intensity treadmill	Attenuated apoptosis, H_2_O_2_ emission and permeability transition pore	Elevated cognitive function and neurogenesis	(101)
Treadmill exercise	Increased TFAM	Decreased the expression of BAD and BAX, increased the expression of BCL-2	(102)
Treadmill exercise	Inhibited mitochondrial outer membrane permeabilization	Reduced neurobehavioral scores and cerebral infarction volumes in stroke model	(103)

## Conclusion

**Figure 1:**
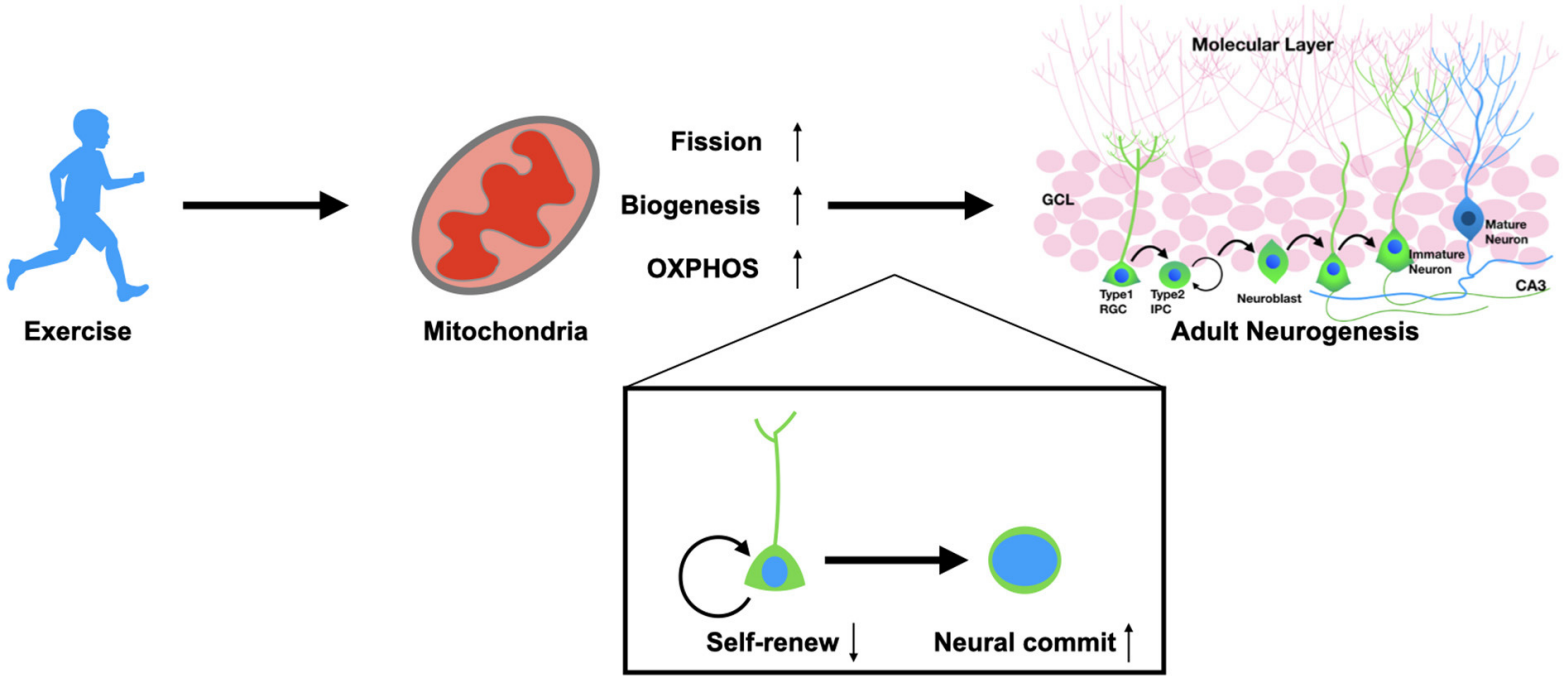
Exercise promotes adult neurogenesis *via* mitochondria. Physical exercise could enhance mitochondrial functions in adult neural stem cells. Exercise promotes the fission of mitochondria, biogenesis, and OXPHOS metabolism. These alterations result in the fate determination of NSCs from self-renewal to neural commitment, which thereby promotes adult neurogenesis in both physiological and pathological conditions.

Mitochondria are key organelles in the mediation of energy functions. Based on this mechanism, recent studies have demonstrated that mitochondria mediate multiple cellular behaviors that are far beyond energy supply, e.g., the fate commitment and proliferation of somatic stem cells as well as the reprogramming and differentiation process of pluripotent stem cells (104, 105). Improving mitochondrial function has also been considered a therapeutic strategy against neurological diseases. Therapeutic approaches targeting mitochondria should focus on future pre-clinical exploration for treating neurodegenerative and psychiatric disorders. Mitochondria play the critical roles in regulating stem cell behaviors including self-renew and fate commitment of the adult NSCs (Figure 1). Therefore, a systematic strategy to improve mitochondrial functions throughout the body is preferable; we should not only promote neuronal regeneration but also focus on regulating the NSCs environment, including the peripheral factors and the neurogenic niche. With such requirements, exercise is the ideal option, accompanied as it is by considerable healing effects and relatively few safety issues.

## Author contributions

All authors listed have made a substantial, direct, and intellectual contribution to the work and approved it for publication.

## Funding

This work was supported by National Natural Science Foundation of China (32000835), and Open Research Fund of the State Key Laboratory of Cognitive Neuroscience and Learning (CNLZD2104).

## Conflict of interest

The authors declare that the research was conducted in the absence of any commercial or financial relationships that could be construed as a potential conflict of interest.

## Publisher's note

All claims expressed in this article are solely those of the authors and do not necessarily represent those of their affiliated organizations, or those of the publisher, the editors and the reviewers. Any product that may be evaluated in this article, or claim that may be made by its manufacturer, is not guaranteed or endorsed by the publisher.
